# Gene Therapy for Heart Failure

**DOI:** 10.1007/s12265-025-10736-6

**Published:** 2026-02-05

**Authors:** Hanna Wang, Anni Määttä, Seppo Ylä-Herttuala

**Affiliations:** 1 A.I. Virtanen Institute, University of Eastern Finland, Kuopio, Finland; 2 Heart Center and Gene Therapy Unit, University Hospital, Kuopio, Finland

**Keywords:** Heart failure, Gene therapy, Therapeutic angiogenesis, Calcium ion cycling, Animal model, Clinical trial

## Abstract

Heart failure is the leading cause of hospitalization globally, burdening healthcare systems and the economy. Heart failure is a multifactorial cardiac syndrome, where the heart fails to maintain sufficient cardiac output to support body function. As the population ages, heart failure rates will increase. Current treatments, such as medications and surgery, are not suitable for all patients, creating a need for alternative therapies. Gene therapy offers promising new approaches, with therapeutic angiogenesis, regenerative strategies, and calcium ion regulation as key targets. SERCA2a plays a critical role in calcium regulation, and clinical trials have focused on its potential as a therapeutic agent. VEGF-B, which specifically targets the myocardium, also regulates myocardial metabolism and SERCA2a activity. Although clinical trials have been conducted, results have not consistently replicated pre-clinical success. This review summarizes the current state of gene therapy for heart failure, including therapeutic agents, vectors, delivery methods, and preclinical models.

**Graphical Abstract:**

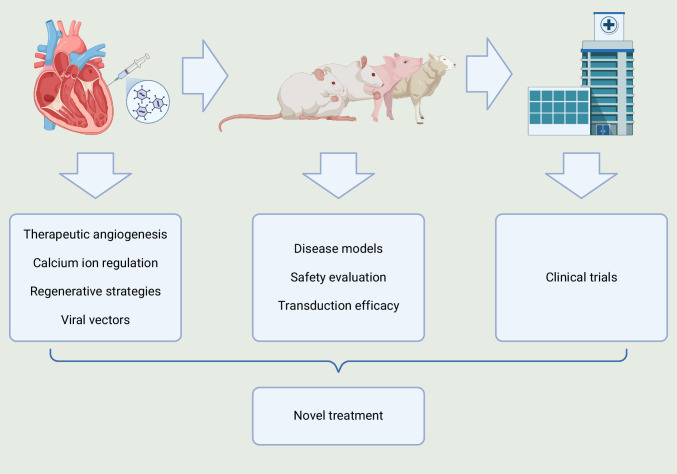

## Introduction

Heart failure is a growing global health problem that burdens both healthcare and the economy [1, 2]. It is a clinical syndrome caused by a functional abnormality in the heart that results in elevated intracardiac pressure and/or inadequate cardiac output. This leads to cardiac symptoms during exercise or in advanced stages also at rest [3–5]. As the average age of the population increases, so does the incidence of heart failure [1], which is the most common reason for hospitalizations in the Western world [6]. The mortality rate of heart failure patients is high with the 5-year mortality rate after hospitalization being approximately 60% [7, 8].

The etiology of heart failure is multifactorial. Typically, heart failure is the result of myocardial dysfunction, which is caused by diastolic or systolic disorders, sometimes by both. In systolic heart failure the heart muscle does not contract efficiently. In diastolic heart failure the heart muscle fails to relax normally between the heart beats. Also, inappropriate function of heart valves, changes in the pericardium and endocardium, and cardiac malformations or arrhythmias, such as atrial fibrillation can cause heart failure [5, 9]. Patients with heart failure are frequently classified by the left ventricular ejection fraction, which can be reduced, preserved or in the mid-range. In the reduced heart failure (HFrEF) the left ventricular EF is under or equal to 40%, in the mid-range (HFmrEF) EF is from 41 to 49% and in the diastolic form (HFpEF) EF is more than 50% [5].

Currently, the treatment of heart failure is based on medication, lifestyle changes and for selected patients pacemakers or invasive procedures. Treatment aims to alleviate the symptoms and improve the prognosis of the patient, mainly by reducing the load on the left ventricle and suppressing neuroendocrine activation triggered by the heart failure. Medications that improve the prognosis of HFrEF are SGLT2-inhibitors and neuro-hormonal antagonists consisting of angiotensin-converting enzyme inhibitors (ACEI), mineralocorticoid receptor antagonists (MRAs) and beta-blockers. Diuretics and digilatis can also be used to reduce symptoms. Digitalis is used for HFrEF patients when they have a high-rate atrial fibrillation [10]. Nevertheless, conservative treatments do not restore damaged heart muscle. Also, in many patients, heart failure progresses beyond optimal conservative therapy. In more severe cases, surgery is required to open occluded coronary arteries or to replace heart valves. Patients with congestive heart failure can be treated with biventricular pacing therapy. Ventricular assistance devices can be used as a bridge to heart transplantation or as a treatment in lieu of the transplant. The only curative treatment is a heart transplant, which is rare in clinical practice [5]. Heart failure is a growing health problem, which still has a poor prognosis despite the improvements in pharmacological treatments. The need for new therapies is vitally important [4].

### Therapeutic Angiogenesis

**Fig. 1:**
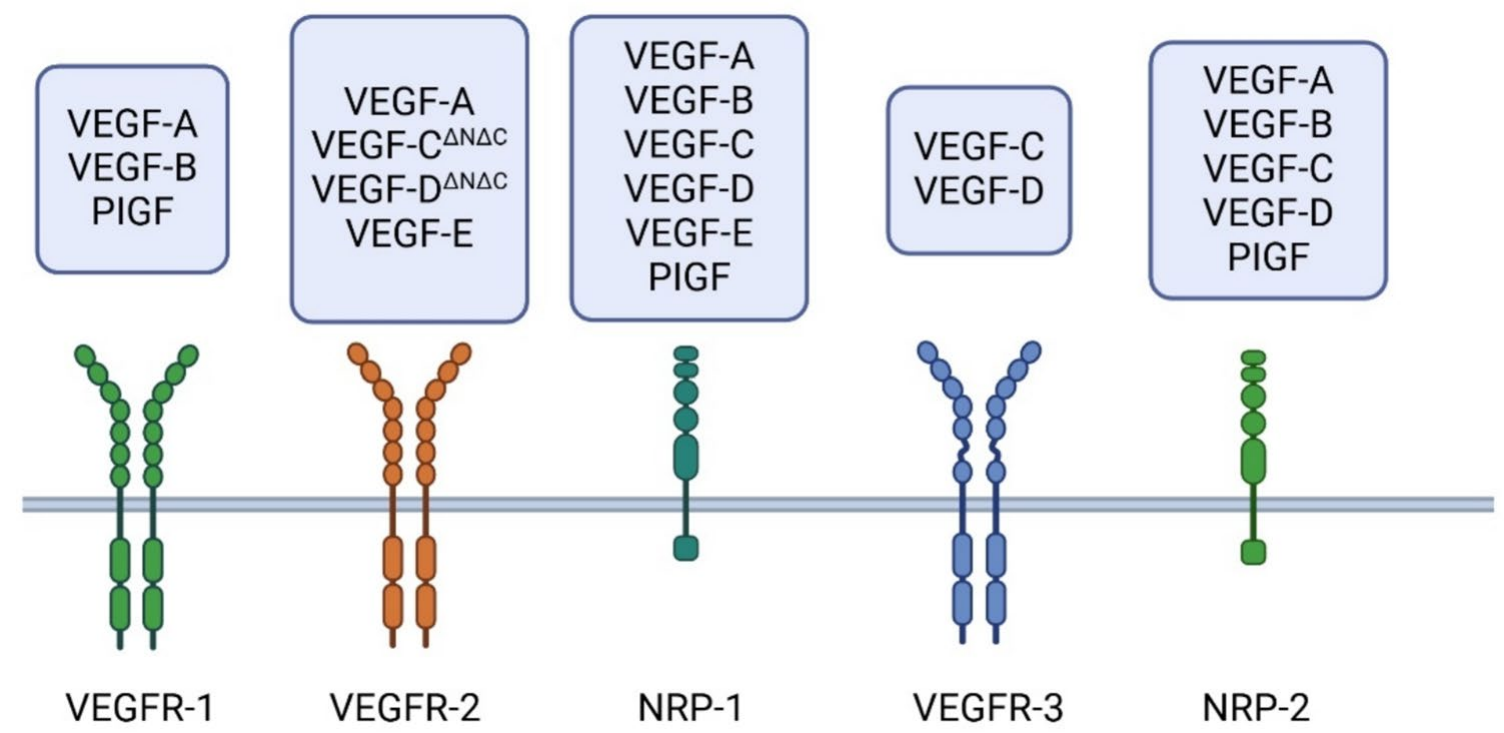
Ligands for vascular endothelial growth factor receptors and co-receptors. VEGFR-1 and VEGFR-2 are mainly expressed on vascular endothelial cells, while VEGFR-3 is widely expressed in lymphatic endothelial cells. Nrp-1 is expressed in many cell types such as neurons, vascular endothelium, and heart, whereas Nrp-2 is primarily found in the lymphatic endothelial cells. Created in BioRender [16]

Therapeutic angiogenesis could become a future treatment for heart failure. In angiogenesis new blood vessels grow from pre-existing vessels. Vascular endothelial growth factors (VEGFs) are very promising transgenes for the therapeutic angiogenesis. VEGF family includes VEGF-A, VEGF-B, VEGF-C, VEGF-D, and placenta growth factor (PIGF). VEGFs signal by binding to tyrosine kinase receptors VEGF-R1, VEGF-R2 and VEGF-R3 and co-receptors neuropilin 1 and 2 (NRP-1 and −2) ([11]Fig. 1) [11]. VEGFs can improve perfusion resulting in improvement in the overall myocardial condition which is desirable for heart failure treatment [12]. They also influence energy metabolism [13] and possible stem and progenitor cell recruitment [14, 15] resulting in regenerative changes, especially VEGF-B and VEGF-D. These characteristics make VEGFs attractive in therapeutic settings of heart failure.

VEGF-A has not been widely used for heart failure gene therapy whereas VEGF-B is relatively myocardium-specific, at least in larger mammals, which makes it an interesting target for gene therapy aimed at treating heart diseases [12]. VEGF-B is highly expressed in metabolically active tissues including the heart [13–15, 17]. Currently, two alternative spliced isoforms of VEGF-B have been identified: VEGF-B167 and VEGF-B186 [18], with VEGF-B167 being the predominant isoform accounting for 80% of the VEGF-B transcripts [19]*.* Both VEGF-B isoforms bind to VEGF-R1, NRP-1 and NRP-2, but VEGF-B186 binds to Nrp-1 and −2 receptors only after proteolytic modification [11].

VEGF-B is a cardioprotective growth factor. Besides angiogenesis, it regulates myocardial contractility, metabolism and protects cardiomyocytes from ischemic and apoptotic damages by downregulating the expression of proapoptotic genes [20–22]. VEGF-B167 induces *α-MHC*, *SERCA2a*, *RYR*, *ANF*, *BNP*, and *PGC1α* genes and represses the expression of *β-MHC* and *α-actin 1* genes, which maintains the contractility of the heart muscle [23]. In heart failure the level of VEGF-B is reduced [12, 24]. VEGF-B promotes angiogenesis also in the subendocardial cardiac region [25].

The signaling pathways mediating VEGF-B-induced angiogenic effects have remained somewhat unclear despite extensive studies. It has been suggested that endogenous VEGF-A binds more efficiently to VEGF-R2 when VEGF-B is present, resulting in angiogenesis [20, 26]. Additionally, Lähteenvuo et al. found that VEGF-R1 and NRP-1 receptors are important for VEGF-B-induced angiogenic effects [12]. On the other hand, Korpela et al. showed in their study by using mutated VEGF-B186 isoforms that only the long form of VEGF-B186 (binds only to VEGF-R1) is able to induce angiogenesis, but the shorter isoforms VEGF-B127 (binds to both VEGF-R1 and NRP-1) and VEGF-B109 (binds only to VEGF-R1) are not angiogenic [27]. The results provided by Korpela et al. and Mallick et al. suggest that there are still unknown receptors and/or mechanism that mediate VEGF-B effects [27, 28].

Indeed, Mallick et al. showed that the long form of VEGF-B186 induces endothelial progenitor cell recruitment via the upregulation of proangiogenic and hematopoietic growth factors leading to angiogenesis [11]. Also, Sultan et al. described a distinct VEGF-B-induced endothelial cell population [29]. Recently, RGD-binding integrins were found as novel receptors of VEGF-B186 that can mediate VEGF-B-induced endoplasmic reticulum stress and support angiogenesis [28].

VEGF-B is co-expressed with a cluster of nuclear encoded mitochondrial genes which are involved in the oxidative phosphorylation [30]. Through binding to its receptors, VEGF-B regulates vascular fatty acid uptake by promoting the activity of transport proteins in endothelial cells. Lipid uptake of ECs is also closely related to mitochondrial lipid utilization [30]. Enhancing the VEGF-B signal has shown to interfere with low-density lipoprotein receptor recycling [31]. Excess activation of the fatty acid uptake and utilization in myocardium leads to inhibition of pyruvate dehydrogenase, which is caused by increased mitochondrial acetyl-CoA levels. This leads to glucose oxidation and subsequent glycolysis through several pathways, including insulin signaling [12, 32, 33]. The inhibition of VEGF-B signaling in mice decreased lipid accumulation and improved insulin sensitivity and glucose tolerance [34].

Metabolically active tissues, such as the heart muscle utilize fatty acids as the primary source of energy [35]. Imbalance of the fatty acid uptake and oxidation results in the accumulation of ceramides and diacylglycerols. This affects insulin mediated glucose uptake and related effects on gene expression and signaling in the heart [32, 36–38]. Through regulating fatty acid uptake and oxidation, VEGF-B is involved in mitochondrial dysfunction in the heart [24]. However, there is still uncertainty whether the fatty acid oxidation-related effects were observed due to high-fat diet-induced insulin resistance [39, 40]. Overexpression of VEGF-B in the hearts from transgenic mice showed shifting of the fatty acid oxidation towards glucose metabolism [20] and reduced lipid deposition together with an increased susceptibility to heart failure [41]. Meanwhile, VEGF-B186 overexpression in mouse adipose tissues showed increased insulin sensitivity, glucose tolerance and improved metabolic health [26].

VEGF-D has an essential role in lymphangiogenesis but can also cause strong angiogenesis. VEGF-D binds to VEGF-R2 and VEGF-R3, where the angiogenic effect is signaled through VEGF-R2 [42]. VEGF-D^∆N∆C^ is a proteolytically processed form of VEGF-D, where the N and C terminal ends have been removed and this modification has shown to enhance angiogenicity of the growth factor through increased affinity to VEGF-R2 [42, 43]. VEGF-D^∆N∆C^ has been taken to clinical testing in refractory angina pectoris patients in KAT301 phase 1 trial, which showed promising results after adenoviral VEGF-D^∆N∆C^ gene transfer by improving perfusion reserve in the treated areas after one year follow-up [44–46]. Currently ongoing ReGenHeart phase 2 trial will test its efficacy in a randomized, double-blinded, placebo-controlled multicenter study. Primary endpoints are exercise tolerance and alleviation of symptoms at 6 months follow-up [47].

Stromal cell-derived factor 1 (SDF-1) is a chemokine facilitating tissue repair after injury through stimulation of the anti-inflammatory pathways, vascular density and stem cell guidance to the myocardium [48]. SDF-1 plasma levels can predict the risk of heart failure and mortality [49]. SDF-1 levels increase typically after myocardial infarction, leading to increased amount of cardiac stem cells in the infarct border zone, and improving cardiac function [48]. Expression of SDF-1 induces angiogenesis in both rats and large animals. However, large animal studies failed to improve left ventricular function and reduce infarct size, which was observed in rats [48, 50].

### Therapeutic Targets for Regulating Calcium Ion Cycling

The calcium ion (Ca2 +) cycle plays a crucial role in the ability of cardiomyocytes to contract and relax [51]. Sarcoplasmic reticulum (SR) maintains the intracellular balance of Ca2 + together with SR transport proteins. Abnormality in the Ca2 + handling mechanisms is linked to the development of impaired cardiac contractility and repolarization [52].

**Fig. 2:**
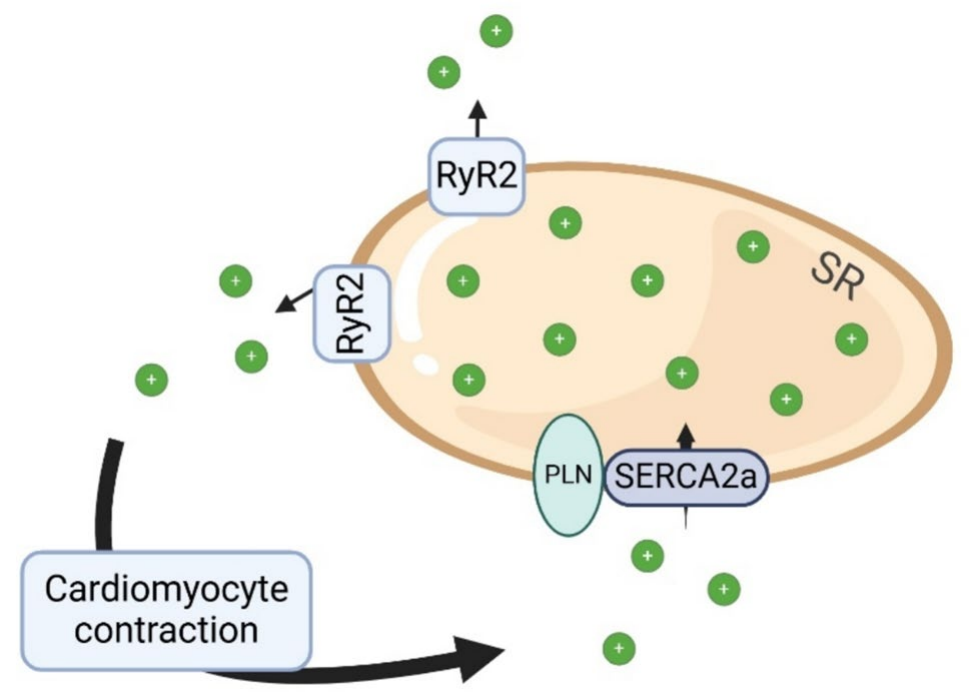
SERCA2a plays a crucial role in Ca2 + homeostasis in cardiomyocytes. During systole Ca2 + ions are released from SR through RyR2 channels. During diastole Ca2 + ions are transported back to SR through SERCA2a. Abnormality in the activity or the amount of SERCA2a is connected to diastolic dysfunction and HFpEF. Created in BioRender [16]

SR ATPase (SERCA) plays a crucial role in Ca2 + signaling by transporting free Ca2 + ions back to the SR (Fig. 2). The main subtype present in the heart is SERCA2a, which functions during diastole [53]. Decreased expression and/or function of SERCA2a has been linked to diastolic dysfunction and HFpEF [54–56], making it an attractive target for therapeutic approaches [57].

Multiple studies in various animal models [55, 58] have shown improvements in cardiac function through enhancing or restoring SERCA2a activity. In a rat model Ad-SERCA2a gene transfer showed improvements in both systolic and diastolic performance in failing hearts as well as in survival and cardiac energetics [58]. The activity of SERCA2a is regulated by several factors, which include phospholamban (PLN), S100A1 and small ubiquitin related modifiers (SUMOs). PLN inhibits SERCA2a activity and phosphorylation of PLN activates SERCA2a through inhibiting the function of PLN [59].

PLN is known to be involved in the development of cardiac diseases, including heart failure [60, 61]. Mutation of arginine 14 in PLN causes severe arrhythmic cardiomyopathy and is relatively common in the Dutch population [62]. In heart failure patients, PLN levels seem to remain stable while PLN phosphorylation and SERCA2a levels decrease causing reduced Ca2 + cycling due to disrupted PLN to SERCA2a ratio [56].

The enhanced activity of type I protein phosphatase (PP1) is associated with reduced PLN phosphorylation in heart failure. Competitive ligands for PP1 have resulted in enhanced PLN phosphorylation in isolated rat hearts [63]. Another way to increase PLN phosphorylation is through overexpressing the constitutively active form of inhibitor 1 (I1c) which acts as an inhibitor for PP1. Enhancing the activity of I1c in a heat failure mouse model resulted in reduced cardiac abnormalities [64].

S100A1 is a calcium-binding protein found in cardiomyocytes regulating Ca2 + related pathways [65]. S100A1 increases SERCA2a activity resulting in improved diastolic and systolic cardiac function as well as cardiac performance both in rodent and swine models [66]. Systemic delivery of a synthetic S100A1 peptide has shown positive results in mouse and pig models [65]. SUMO-1 has shown to regulate the levels of SERCA2a protein, directly affecting the heart function. In heart failure SUMO-1 is decreased, while the introduction of SUMO-1 to heart failure models showed an increase in cardiac function and SERCA2a activity [67–69].

Adenosine 3’,5’-monophosphate (cAMP) regulates multiple cellular responses and intracellular functions through βAR pathway [70]. cAMP levels are lower in heart failure [59, 66]. Although manipulating cAMP-dependent signaling improved cardiac function, it showed signs of undesirable long-term effects on heart rate and energy homeostasis [66, 70]. Adenylyl cyclase (AC) is a group of enzymes that contribute to the synthesis and degradation of cAMP [70]. AC6 is the cardiac isoform of AC [59]. Deletion of AC6 results in the reduction of PLN phosphorylation and SERCA2a activity [70] and overexpression leads to increased LV contractility, improvement in cardiac function and survival through improving Ca2 + homeostasis [59, 70]. AC6 gene transfer has shown promising results in treating heart failure both in mouse and swine models [71, 72].

Plakophilin-2 (PKP2) gene encodes a protein found primarily in cardiomyocytes. PKP2 mutation has been observed in patients with an early onset arrhythmogenic right ventricular cardiomyopathy (ARVC). However, these individuals also have a higher left ventricular ejection fraction. Additionally, ARVC patients with PKP2 mutation presented less frequent left ventricular damage and heart failure [73]. A mouse model with PKP2 deficiency resulted in arrhythmogenic cardiomyopathy with the right ventricular predominance. The loss of PKP2 expression also impacted the expression of genes that maintain Ca2 + homeostasis [74].

### Other Therapeutic Approaches for Heart Failure

There is a growing interest in microRNA (miRNA)-based modulation of heart failure pathogenesis. The expression of several miRNAs is altered in heart failure leading to either pathogenic or cardioprotective roles [75]. For example, miR-25 acts as a suppressor for SERCA2a. In murine heart failure model, the antagonist of miR-25 has restored calcium cycling and improved contractility [76]. On the other hand, restoring downregulated cardioprotective miRNAs like miR-1 or miR-133 could result in reduced hypertrophic growth and arrhythmogenesis [77].

Fibrotic remodeling is an emerging target for progressive heart failure. Recent studies utilize engineered CAR-T cells or mRNA-based reprogramming to either eliminate or modulate activated cardiac fibroblasts [78, 79]. Transient delivery of modified mRNA (modRNA) for acid ceramidase or IGF1 has shown to reduce fibrosis and preserve ventricular function in post-ischemic models [80, 81].

Additionally, in vivo base and prime editing, have enabled precise correction of pathogenic mutations in cardiac regulatory genes. Base editing of CaMKIIδ to prevent pathological oxidation has preserved calcium handling and reduced infarct size in ischemia/reperfusion murine models [82, 83]. These gene editing platforms, while still in preclinical stages, hold promise for long-term correction of disease-driving mechanisms in heart failure.

### Viral Vectors

Viral vectors are used to deliver genetic material to the target tissues. The obvious benefit comes from their ability to transduce cells and transfer genetic material to the host cells. The most common viral vectors are adenovirus (Ad), Adeno-associated virus (AAV) and lentivirus (LV).

Ads were among the first viruses used as a gene delivery vector. Ad vectors allow an efficient delivery of transgenes which translates into an efficient transgene expression. Ads also have a wide tissue tropism which allows targeting a variety of therapeutic targets. Ads do not integrate genetic material into the host genome, reducing the risk of mutagenesis [84]. However, this also shortens the time of transgene expression to one to two weeks. The challenges faced with using Ads mostly consist of its immunogenicity, which poses challenges in achieving efficient treatment responses [75, 85]. The human Ad serotype 5 (Had5) is the most widely studied in both preclinical research [76–78] and in clinical trials [79, 80]. Although the engineering of Ad genome has enabled a large capacity to deliver genetic material to the maximum of 36 kb [81], Had5 is mostly used with its 8.2 kb packaging capacity [82, 86].

AAV is smaller than Ad and dependent on Ad or other viruses to replicate. AAVs are not known to cause human diseases. Due to the small genome size of 4.7 kb the size of the transgene needs to be within this capacity [83]. AAV vectors have a wide tissues tropism making them as useful vectors for a range of diseases [87–89]. AAV genomes can recombine to a stable and persistent configuration that can stay detectable for several years. However, this raises concerns on the long-term safety and the risk of mutagenesis [90]. AAVs are less immunogenic than Ads although they still pose challenges in patients that have previously encountered AAVs. These patients are very likely to develop neutralizing antibodies and T-cell responses against the vector capsid [91].

The choosing of a serotype for testing is influenced by the natural tropism of the virus and on the prevalence of neutralizing antibodies in general population. AAV1, AAV6 and AAV9 have the highest tropism towards cardiac tissues [92]. AAV6 has been used in multiple heart failure models [93–95] and AAV1 and AAV9 have shown to be well tolerated in humans [96, 97]. AAV6 and AAV9 show strong expression in animal models of heart failure. AAV6 has shown only low pre-existing immunity, making it an attractive serotype for clinical use. New modified serotypes are being generated to address challenges concerning pre-existing immunity. Recombinant AAV capsids are genetically engineered vectors created to enhance tissue specificity, improve transduction efficiency and evade pre-existing immunity [98].

LVs enable for a long-term therapeutic effect since they integrate the transgenic into the host genome [99]. The packaging capacity is roughly the same as in Ad [100]. Depending on the vector design, LVs can present relatively weak immune responses [101]. To limit the risk of mutagenesis, multiple advances in the vector design have been taken. Self-inactivation and insulators are added to the vectors to reduce the risk of insertional mutagenesis and activation of nearby genes [102].

### Gene Transfer Methods

Over the years different gene transfer methods have been considered for heart failure therapy. Because of the physiology and size differences rodent models have proven not to be the most useful tools for clinical development. Instead, large animal models have helped in the development of clinically relevant methods. The most used approaches can be simply divided into two main categories: myocardial injections and intravascular infusions.

For myocardial injections both epicardial and intramyocardial approaches have been used. Epicardial injections are introduced via thoracotomy and therefore require a highly invasive procedure. Intramyocardial injections on the other hand can be performed minimally invasively while achieving a similar transduction efficiency [46, 86, 103]. However, targeting the injections to the target area is much more challenging compared to the epicardial injections. To overcome this limitation navigation systems have been developed to visualize the desired target areas [104, 105]. The limitation of myocardial injections in the context of heart failure is the restricted area of effect and the limited number of injections [106].

Intravascular approaches utilize coronary arteries or veins for vector delivery, which can be categorized as antegrade or retrograde methods. Intravascular methods allow for a broad area of vector distribution, making it potentially ideal for the treatment of heart failure [106]. In retrograde infusion procedures the operator would need to catheterize GCV, LMV and ACV to achieve a global effect of the gene transfer. During the gene transfer the corresponding coronary artery can be blocked temporarily while the vector is infused through the catheter placed in the corresponding coronary vein [107]. The overall catheterization procedure requires skill, patience and experience. Of course, any blocking of the coronary vasculature during the operations carries a risk of developing ischemic damage. A downside to a broad distribution is the unwanted vector distribution to other organs [108]. However, improvements are being made to develop more specific vectors able to target myocardium as efficiently as possible.

### Preclinical Animal Models

Animal models are needed to ensure safety and feasibility of novel treatments before applying them to human patients. Animal model-based research has provided insights into disease mechanisms leading to the development of new treatments. Of course, no animal model is perfect for predicting the outcomes in humans but through carefully planned and implemented studies a lot can be learned from these experiments in well controlled environments.

Smaller animals are cost-effective and time-efficient, making them suitable for preliminary testing. Mice and rats are easy to maintain, as well as having short gestation periods and lifespans. Cost-effectiveness allows for larger group sizes and shorter lifespan has allowed for creation of different strains and genetically modified models. Small animal models are good for proof of principle studies. However, there are several limitations in comparison to humans. Physiologically small animals have much higher heart rates (mice 300–840 bpm, rat 330–480 bpm), different metabolism and are nocturnally active.

Large animal models are anatomically, hemodynamically and physiologically much more like humans. With increased size, the animals are more expensive. Genetically modifying large animals is much more challenging because the litter sizes are usually much smaller, and the long gestation period slows down effective reproduction. The most used models for cardiovascular research are porcine and ovine models [92]. Pigs are susceptible to tachyarrhythmias and sudden death [93]. They also tend to gain weight rapidly. Sheep on the other hand stay at a manageable weight allowing for longer follow-up periods, but their thoracal anatomy and a habit of sleeping on their sternum makes sternotomies unpractical. Non-human primates have been increasingly used [94] as models of human diseases despite the overall decline in the support for primate use in the scientific community. The obvious advantage in using non-human primates is the high similarity to humans both genetically and anatomically. However, ethical considerations can be complex not to mention the high cost of maintenance [95]. Despite the close similarity to humans, non-human primate models are not frequently used in heart failure research [96].

In addition to genetically modified animals, several different models have been developed for heart diseases. Roughly, the methods can be divided into surgical interventions, pressure, volume or pacing overloading, and drug induced methods [96]. Surgical methods involve ligating terminally or occluding temporarily left anterior descending artery (LAD) to model myocardial infarction. In small animals it is also possible to ligate coronary arteries via popping out the heart through thoracotomy [97]. In large animals the occlusion can be made using catheter-based techniques resulting in lowered invasiveness and risk of infections. The idea behind these surgically created models is to simulate an ischemic environment, which is a known precursor for heart failure. Pressure overloading models are made through manipulating the aorta [96]. In pressure overloading models the idea is to mimic the effect of chronic hypertension, which is a major contributor to heart failure progression. Volume overload models are more commonly used in larger animals since they involve the need to access cardiac valves [96].

Like volume overload, rapid pacing is mostly restricted to large animals for the fact that placing electrodes in rodent hearts is very challenging and they already have a high heart rate. In large animals, a standard pacemaker can be used to create heart failure models with different severity. Over pacing of 220 to 260 bpm has been shown to result in the development of lowered cardiac output and a subsequent biventricular heart failure in three weeks [98]. To achieve a more consistent development the pacing can be as low as 120–200 bpm to result in severe heart failure in four to eight weeks [109, 110].

### Clinical Trials for Heart Failure

Several preclinical trials with promising results have proceeded to clinical trials (Table 1). CUPID2, AGENT-HF and SERCA-LVAD are randomized placebo-controlled studies, which used intracoronary infusion as a delivery method and AAV1 as a vector with the same dose. CUPID2 had 250 patients with severe heart failure, whereas SERCA-LVAD targeted patients with a left ventricle assistance device and AGENT-HF targeted 10 patients with congestive heart failure. In CUPID 2, the primary endpoint was the recurrence of clinical events, such as reduction in mortality and HF-related hospitalization. SERCA-LVAD evaluated the safety and feasibility of gene therapy and AGENT-HF examined the gene transfer efficacy on the left ventricular end-systolic volume. CUPID2 has already produced the main results from Phase 2 -trial. Unfortunately, CUPID2 results showed no improvement in the primary endpoints, leading to the termination of AGENT-HF and SERCA-LVAD trials. The reason behind negative results, is not completely clear. Speculation on changes in manufacturing, resulting in poorer AAV1 products or neutralizing properties of pre-existing antibodies resulting in ineffective delivery are suggested. However, CUPID2 demonstrated the safety of AAV1/SERCA2a gene therapy [112, 113].

**Table 1:** Recent clinical trials on heart failure gene therapy

Trial	Vector	Therapeutic agent	Delivery	Dose	Study design	*n*	Primary Endpoint	Year	Current satuts	Main results	Identifier
-	Plasmid	VEGF165	intramyocardial injections	N/A	PHASE1, Open label	12	LVEF measured by echocardiogram	2007	WITHDRAWN	-	NCT00279539
-	Ad5	Hac6	i.c. infusion	3.2E9vg to E12vg	PHASE1|PHASE2, RCT	56	Excercise treadmill time, LV function by echocardiography, rate of LV pressure	2009	COMPLETED	N/A	NCT00787059
-	Plasmid	ACRX-100 (SDF-1)	intramyocardial injections	N/A	PHASE1, Open label	17	Number of MACE at 30 days post-injection	2010	COMPLETED	N/A	NCT01082094
CUPID2	AAV1	SERCA2a	i.c. infusion	3E12vg/1E13vg	PHASE1|PHASE2, RCT	51	Time to recurrent cardiovascular events	2013	COMPLETED	No improvements but safe	NCT00454818
SERCA-LVAD	AAV1	SERCA2a	i.c. infusion	1E13DRP	PHASE2, RCT	5	Safety and feasibility	2014	TERMINATED	N/A	NCT00534703
AGENT-HF	AAV1	SERCA2a	i.c. infusion	1E13vg	PHASE2, RCT	10	Changes in left ventricular end-systolic volume	2014	TERMINATED	N/A	NCT01966887
-	AAV1	SERCA2a	i.c. infusion	2.5E13DRP	PHASE1|PHASE2, RCT	9	Safety profile	2015	TERMINATED	N/A	NCT02346422
-	Plasmid	INXN-4001 (SDF-1α, VEGF165, and S100A1)	retrograde coronary sinus infusion	80 mg in 40 ml/80 ml	PHASE1, RCT	12	Safety and feasibility	2018	COMPLETED	N/A	NCT03409627
FLOURISH	Ad5	Hac6	i.c. infusion	10E12vg	PHASE3, RCT	536	Hospitalization due to heart failure up to 12 months	2019	WITHDRAWN	-	NCT03360448
-	LNP	NTLA-2001 (CRISPR/Cas9)	intravenous dosing	N/A	PHASE1, Open label	72	Treatment-emergent adverse events, clinical laboratory test findings, change in serum TTR or prealbumin and concentration, half-life, distribution of DMG-PEG2k, LP000001, Cas9 mRNA, and sgRNA. Time frame 2 years	2020	ACTIVE	-	NCT04601051
GenePHIT	AAV2i8	AB-1002 (I1c)	i.c. infusion	3.25E13vg/6.5E13vg	PHASE2, RCT	150	Cardiovascular related death, NYHA classification change from baseline, peak oxygen update, LVEF change from baseline, 6-min walk test. Time frame 52 weeks	2023	RECRUITING	-	NCT05598333
MyPEAK-1	AAV9	MYBPC3	introvenous dosing	3E13 vg/kg/6E13 vg/kg	PHASE1B | PHASE 2, Open label	30	Number and severity of Adverse Events over the course of the study. Time frame 5 years	2023	RECRUITING	-	NCT05836259
MUSIC-HFpEF	AAV1	SERCA2a	i.c. infusion	3E13vg/4.5E13vg	PHASE1, Open label	10	Change in pulmonary capillary wedge pressure (PCWP)	2024	RECRUITING	-	NCT06061549
RIDGE-1	AAV9	PKP2	intravenous dosing	3E13vg/kg/6E13vg/kg	PHASE1, Open label	15	Safety, Tolerability, Dose and Pharmacodynamic	2024	RECRUITING	-	NCT06228924

*DPR* DNase-resistant particles, *MACE* Major Adverse Cardiac Events, *LVEF* left ventricle ejection fraction, *RCT* Randomized controlled trial, *N/A* Not available

clinicaltrials.gov [111]

Two other studies investigate the effects of AAV1/SERCA2a therapies using intracoronary infusion. MUSIC-HFpEF, an open-label phase 1 study, evaluates the safety and pharmacodynamic effects of AAV1-SERCA2a in 10 patients with HFpEF. MUSIC-HFpEF had 4 years follow-up time with the primary endpoint being the change in pulmonary capillary wedge pressure. Study nr NCT02346422 consists of open-label phase 1 and randomized, double-blinded and placebo-controlled phase 2 trial. The aim was to evaluate the safety and preliminary activity of the high-dose AAV1/SERCA2a in patients with advanced heart failure. The primary endpoints in both phases were safety and incidence and event rates of hospitalizations.

RIDGE-1 and GenePHIT are also AAV based trials. RIDGE-1, a phase 1 study, evaluates the safety and preliminary efficacy of PKP2 gene therapy in 15 patients with symptomatic PKP2 mutation-associated ARVC. Patients will receive a single intravenous dose of AAV9/PKP2 with two different doses. The aim of the study is to examine safety, tolerability, dose and cardiac transgene expression. GenePHIT trial is a double-blinded, randomized, placebo-controlled, multicenter phase 2 study. The study focuses on 150 patients with nonischemic cardiomyopathy and severe symptoms of heart failure. The therapy is delivered through intracoronary infusion and the safety and efficacy of a single dose of AB-1002 (I1c) will be examined and evaluated versus placebo. GenePHIT trial result should be available approximately in 10/2026.

Currently there are two Ad-based clinical trials. Both trials use intracoronary infusion as the delivery method and investigate the effects of adenylyl cyclase 6 (AC6). The first study investigates whether gene transfer offers therapeutic benefits in patients with congestive heart failure. The primary measurements are exercise treadmill time, LV function by echocardiography before and during dobutamine infusion and the rate of LV pressure development and decline before and during the dobutamine infusion. Flourish is a phase 3 trial and it examines AC6 gene transfer in patients with HFrEF. It is a randomized, placebo-controlled, double-blinded, multicenter trial. The aim of the study was to determine the safety and efficacy of Ad5/AC6. The primary endpoint was hospitalization due to heart failure up to 12 months. LV function was measured by echocardiography at rest and under dobutamine stress. The improvement was seen in the high dose group and gene transfer appeared to be safe. Unfortunately, there were no differences between the AC6 treated and placebo-treated patients in treadmill testing [80]. The trial faced several challenges. The vector enabled short term gene expression, and cardiac uptake was limited. The trial’s focus on clinical endpoints, such as hospitalization and cardiovascular death, demanded more robust and durable efficacy than the therapy could deliver. These factors, combined with the limitations of Ad5 vectors in chronic diseases, led to the discontinuation of the program.

In addition, there are three phase 1 trials which use plasmids as vectors. These studies use either retrograde coronary sinus infusion (RCSI) or intramyocardial injections as the delivery method. The first study (NCT03409627) evaluates the safety of a novel triple-effector plasmid (INXN-4001) in 12 patients with left ventricular assist device. The primary endpoint is adverse events occurring up to 6 months post-RCSI. The second study (NCT00279539) investigates the safety and bioactivity of intramyocardial gene transfer using a VEGF165 plasmid to stimulate new blood vessel growth in the heart, with the goal of improving circulation in areas with inadequate blood flow. The third study (NCT01082094) assesses the safety, tolerability and preliminary efficacy of ACRX-100 in 17 patients with ischemic heart failure. ACRX-100 is a DNA plasmid engineered to transiently express human stromal-cell derived factor 1 (SDF-1). The primary endpoint is the number of major adverse cardiac events.

MyPEAK-1 and NCT04601051 are gene therapy-based clinical trials focused on treating cardiomyopathies which can lead to heart failure. MyPEAK-1 evaluates the safety, tolerability, and pharmacodynamics of AAV9/MYBPC3 gene therapy in 30 adults with symptomatic hypertrophic cardiomyopathy. The study is a first-in-human, non-randomized, open-label study with two dose cohorts. The NCT04601051 trial is a phase 1, open-label study assessing the safety, tolerability, pharmacokinetics, and pharmacodynamics of NTLA-2001 in 72 patients with hereditary transthyretin amyloidosis with polyneuropathy (ATTRv-PN) or transthyretin amyloidosis-related cardiomyopathy (ATTR-CM). This trial utilizes CRISPR-Cas9 gene editing, delivered via lipid nanoparticles, to achieve in vivo knockout of the mutated transthyretin (TTR) gene. Both trials utilize intravenous delivery and the follow-up times are five years in MyPEAK-1 and two years in NCT04601051.

### Key Points Learned from Clinical Trials

Unfortunately, many successful pre-clinical approaches have not been effective in clinical trials. The efficiency of gene therapy is determined by several factors. A suitable vector must be selected to deliver the desired gene to the target cells. Gene therapy depends on the level of the transgene expression in the target cells. Expression is affected by the chosen vector, dose of the transgene, the gene delivery method and the interaction between the host cells and the vector [114]. Also, immune responses to the vector and the transgene may affect the results [115]. Despite the poor efficacy in clinical trials, several trials have established a good safety profile of these novel therapies [44, 103, 116, 117]. One key aspect in the future trials will be the identification of patient subgroups who are most likely to benefit from the new therapies. Gene therapies have great placebo effects. Thus, the studyies benefit from double-blinded and randomized trials designs.

### Gene Delivery Methods

Gene delivery into the myocardium has been a major challenge. Heart failure requires treatment of the majority of the ventricular cardiomyocytes, which is challenging. To be successful, gene transfer should be efficient and targeted as globally as possible into the heart muscle. Also, minimal off-target gene expression would be desired [118]. Gene transfer will not achieve biological effects if the concentration or the persistence of the therapeutic proteins are not sufficient [119]. Of the present gene delivery methods the intramyocardial injections and retrograde intravenous delivery methods appear to be the most effective for the treatment of heart failure [107, 120].

Catheter-based intramyocardial injections have been proven safe [46]. One of the strengths of the intramyocardial method is the precise targeting of the injections. However, the diffusion area of the viral vectors in the myocardium is limited. In one hand this adds to the safety and locality of the treatment but on the other hand limits the treatment from reaching the whole heart muscle which would be the overall target in heart failure. Intramyocardial injections can cause mechanical stress on the cells during injections which may impair effectiveness [121]. Intramyocardial delivery during cardiac surgery might be the most controlled way to do the gene transfer [118]. However, with heart failure patients, anesthetic and other operative risks should be considered.

Intracoronary delivery can theoretically result in a global transduction compared to myocardial injections. However, antegrade delivery has been shown to have a low efficacy when tested in humans [112] and intravascular gene therapy methods may be challenging in patients with coronary artery occlusions. Retrograde coronary vein infusion has shown a global transduction efficiency and promising results in large animals [107, 120]. With any intravascular method, there is a risk of the release of the vector to systemic circulation which affects non-target tissues. Also, potential activation of the immune system may reduce the effectiveness of intravascular gene therapy [118]. Total global delivery is still challenging, but perfusion methods and retrograde delivery are probably the most effective strategies.

### Vectors

To achieve clinically relevant treatment effects the vector should target gene expression to cardiomyocytes, escape the immune system and maintain long enough transgene expression [118]. However, for some transgenes the expression time should not be too long since powerful factors like VEGF will be detrimental to tissue architecture [119].

The efficiency of gene transfer could be improved by paying attention to a few points related to the vectors. Immune barriers, such as neutralizing antibodies and innate or adaptive immune system responses may limit the therapeutic effect. This could be reduced by testing anti-vector antibodies before gene transfer and selecting the vector type according to the immunological status of the patients [119]. In cases needing repeat dosing, changing the serotype of virus should be considered to avoid neutralizing effects. Using vectors with a better cardiac tropism should increase the effect in the heart. [66, 122–125]. In the future, it might be possible to develop vectors with better cardiac-specific promoters and where transgene expressions could be regulated by small molecules or physiological stimuli [126]. Also, the efficient dose of the vectors is still unclear. In preclinical animal models the vector doses have usually been much higher than in human trials [127]. However, only very few clinical trials have tried to demonstrate actual gene transfer efficiency in human target tissues [128, 129]. There is a clear need to develop new vectors that can escape innate and adaptive immunity, have high tropism to the heart muscle and have long enough transgene expression time.

### Patient Selection

Currently non-controlled and non-randomized gene therapy trials in cardiovascular diseases have provided positive outcomes, while most randomized, controlled and blinded studies have not reached any clinically relevant effects in the heart muscle [118]. In many previous trials, the patients selected have been so called “no option” patients who have varying comorbidities. In the future, healthier patients should be recruited to trials because these “no option” patients have usually lost at least some of their regenerative capacity. Therefore, they are not optimal to test new therapeutic approaches [121]. It is also possible that only some subgroups of patients will respond positively to gene therapy approaches [118]. It would be essential to study biomarkers or other characteristics to specify the most optimal patients for clinical trials [46, 130].

### Endpoints

In cardiovascular gene therapy, selecting appropriate endpoints is crucial for evaluating therapeutic efficacy and safety. Heart failure develops gradually with different grades of disease manifestation and complications. Usually, late manifestations are treated with gene therapy which means that treated patients often already have complicated diseases, medications and aging-related issues [131, 132]. Traditional endpoints, such as survival rates, hospitalization and exercise tolerance are often too demanding for chronically ill, aged individuals and may not fully capture the therapeutic potential of novel treatments [118]. Therefore, there is a need to develop validated surrogate endpoints that mimic meaningful clinical improvements.

Potential surrogate markers could include tissue perfusion, collateral blood flow, metabolic enhancements, and a reduced need for hospital services. Implementing these surrogate endpoints can provide a more nuanced understanding of the therapeutic impact [132, 133]. Imaging-based assessments, such as PET, MRI and ultrasound provide precise and quantifiable measurements of tissue perfusion, blood flow, and metabolic activity, making them ideal for assessing the effects of gene therapy. These objective measurements are less subjective than traditional endpoints like exercise tolerance or quality-of-life questionnaires, providing a clearer picture of the real-world impact of the treatments [118]. Also, these measures would be applicable both in clinical and preclinical studies.

It is essential to measure the presence and activity of the therapeutic proteins or RNA before claiming any therapeutic effects. Without this demonstration, attributing observed effects to gene therapy is questionable. To ensure that the clinical results are truly reflective of the gene therapy, it is crucial to design and validate surrogate markers for both transduction efficacy and biological activity of the gene therapy product [118]. Additionally, immune responses and pre-existing antibodies are crucial factors for the outcomes of gene therapy. The presence of pre-existing antibodies to viral vectors used in gene delivery impairs effectiveness by neutralizing the vector or triggering immune responses that limit therapeutic benefits. Thus, evaluating these immune factors before and during gene therapy is essential [133].

Addressing these challenges involves developing appropriate endpoints, including advanced imaging techniques, and ensuring that both preclinical and clinical studies based on translatable and clinically meaningful measures. The inclusion of biomarkers, as well as an understanding of the immune responses and pre-existing antibodies, further enhances this approach, contributing to more accurate and reliable assessments. Such efforts are crucial for advancing the field of cardiovascular gene therapy and achieving successful clinical outcomes.

### Animal Models and Human Heart Failure

It is very challenging to replicate multifactorial diseases in experimental animals and usually disease models are much simpler. Unfortunately, this would also mean that the tissue environment is different, which affects the therapeutic outcome. For example, entry of the viral vectors into the target cells, capacity to produce transgenes, cell types that respond to therapeutic molecules and ability for regeneration need to be considered [134].

Another aspect to account for is the notable differences between animals used for research and human patients. Laboratory animals are often young, which is why they naturally have a better regenerative capacity. Also, environmental variables, such as dietary factors and circadian rhythm can be easily harmonized and closely controlled [135, 136]. Additionally, animal models lack exposure to some factors that humans may encounter during life, such as exposure to nicotine or alcohol. These factors minimize differences between laboratory animals, so that even small differences between the groups can lead to significant results.

**Fig. 3:**
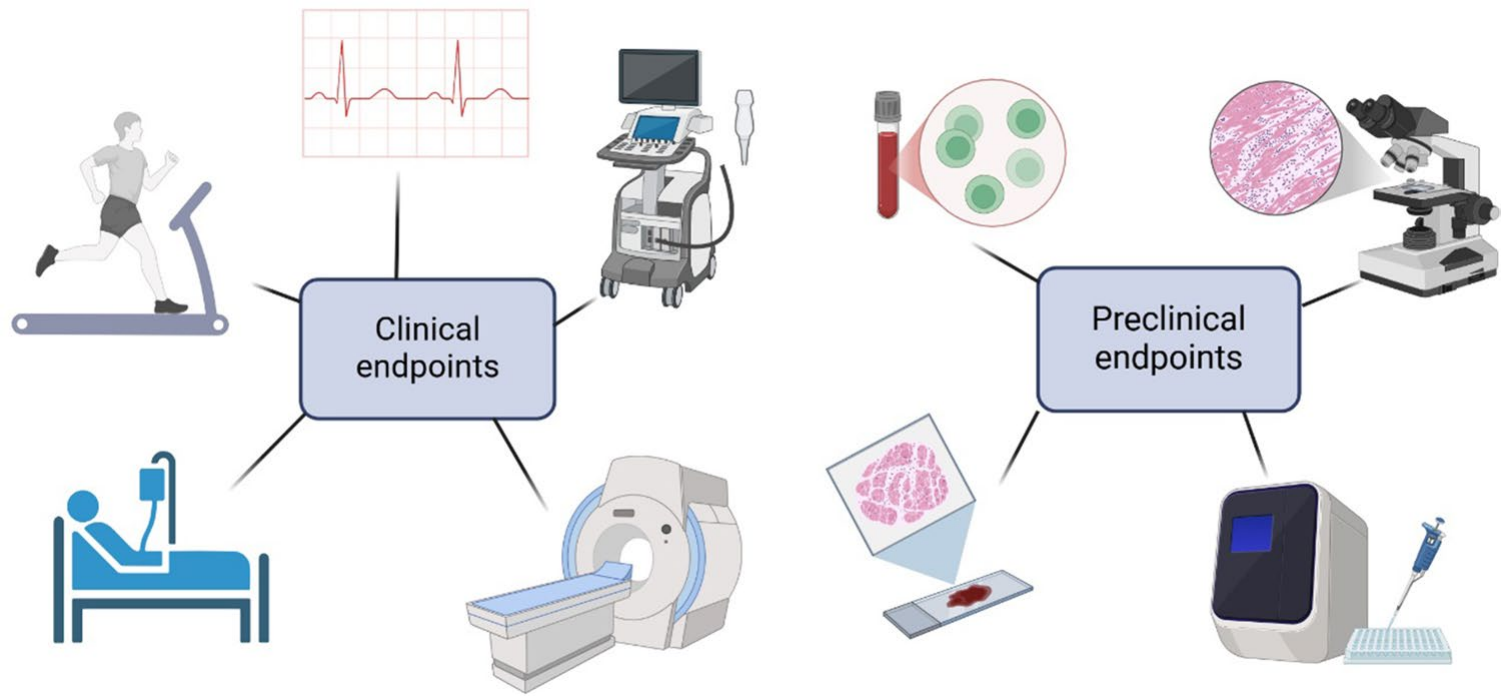
The success of gene therapy depends on carefully selected endpoints that accurately reflect its effects. Traditional clinical endpoints, such as survival rates, may not be suitable for chronically ill patients, making imaging techniques more precise alternatives. Additionally, assessing therapeutic protein expression and immune responses is crucial. Differences between animal models and human patients must be considered to ensure reliable research outcomes. Thorough evaluation supports the advancement of gene therapy into clinical applications. Created in BioRender [16]

**Fig. 4:**
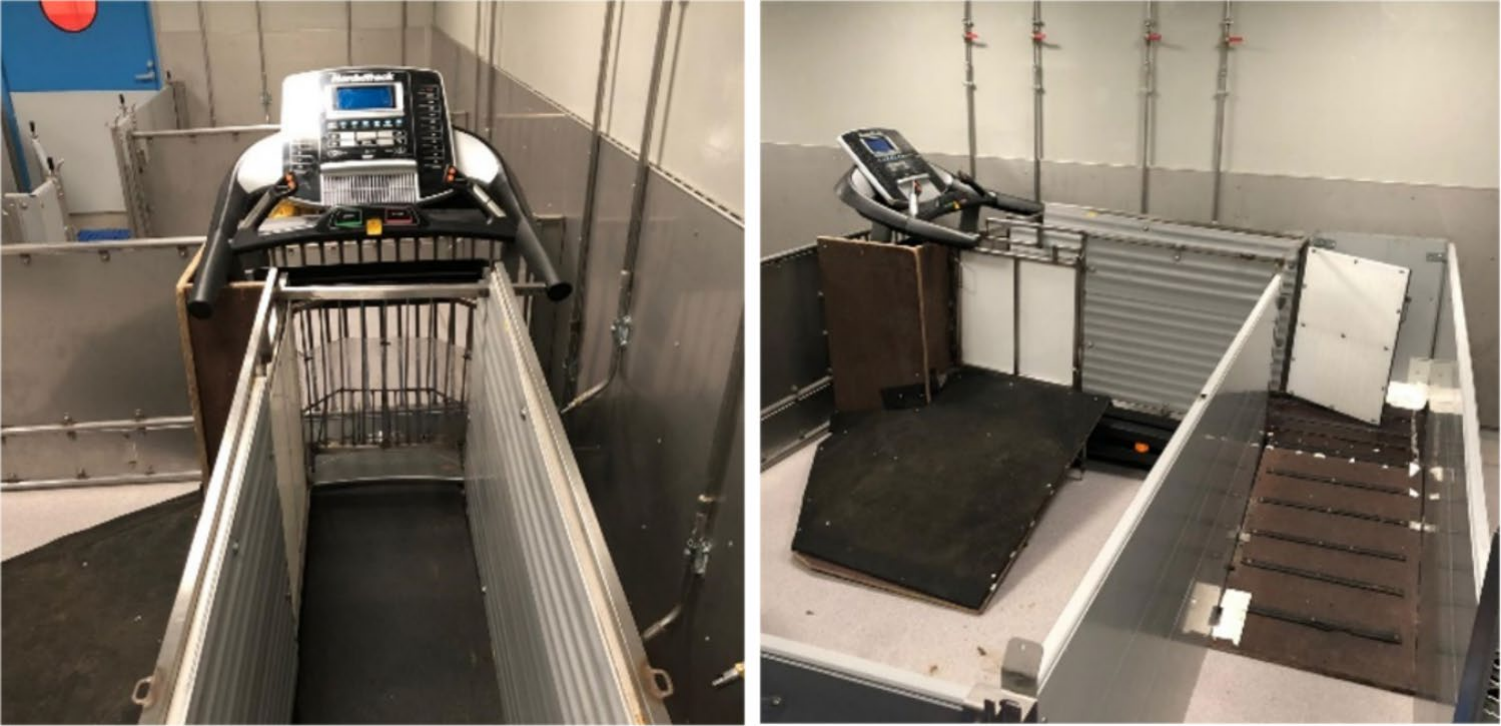
Functional measurements, such as exercise test, provide valuable information about the effects of treatment. Especially in large animal models, the exercise test can be performed on a treadmill [137]

One explanation for a poor success in clinical trials is a mismatch between endpoints (Fig. 3). In pre-clinical studies, the treatment target is often one molecule and focuses on one organ, which limits the scope of the research. However, heart failure in human patients presents as a multifactorial syndrome. Together these factors create clinical manifestations, and different molecular events can lead to similar clinical symptoms. Typically, preclinical trials focus on quantifiable endpoints, for example the number of capillaries or the amount of gene expression. In clinical trials, the success of the treatment is often evaluated by subjective endpoints, such as quality of life or exercise tolerance [132]. An increased number of blood vessels does not automatically improve the contractility of heart muscle in heart failure [118]. Functional endpoints should be added to preclinical studies because the main goal is always to alleviate patient syndromes (Fig. 4) [137]. However, molecular information in preclinical studies provides important information about therapeutic molecules, but not from the symptom-based treatment.

The success of preclinical and clinical trials requires comprehensive knowledge of the advantages and disadvantages of gene therapy. Despite the challenges, gene therapy has progressed as a form of treatment and the first gene drugs have been approved for clinical use in Western countries [137, 138].

## Conclusions

Gene therapy presents a promising option for the treatment of heart failure, offering potential solutions where conservative treatments are not sufficient. Despite significant advancements in preclinical models, translating these findings into successful clinical applications remains a challenge. Key challenges include optimizing gene delivery methods, selecting appropriate vectors, and identifying patients who would benefit most from such treatments [134].

So far, only a limited number of clinical trials have been conducted to evaluate the efficacy of gene therapy on heart failure. CUPID2, one of the most extensive trials in the field, demonstrated the safety of gene therapy but failed to show significant clinical benefits in reducing hospitalizations or mortality [112]. These results highlight the complexity of gene therapy and the need for further research to refine patient selection, optimize dosing strategies, and improve targeting and control of gene expression. To date, there is no gene therapy approved for heart failure.

While clinical trials have demonstrated the safety of gene therapy, efficacy in improving long-term cardiac function is still missing. Future research should focus on refining vector technology, enhancing the specificity of gene expression, and developing patient-specific therapeutic strategies. With continued innovation, gene therapy has potential to revolutionize the treatment of heart failure and to improve patient outcomes in severe heart diseases.

## Data Availability

This review did not produce any new data.

## Abbreviations

HFrEF: Heart failure with reduced ejection fraction
HFmrEF: Heart failure with mid-range ejection fraction
HFpEF: Heart failure with preserved ejection fraction
ACEI: Angiotensin-converting enzyme inhibitors
MRA: Mineralocorticoid receptor antagonists
VEGF: Vascular endothelial growth factors
PIGF: Placenta growth factor
VEGF-R1, -2 and -3: Vascular endothelial growth factor receptors 1, 2 and 3
NRP-1 and -2: Neuropilin 1 and 2
SDF-1: Stromal cell-derived factor 1
Ca2 +: Calcium ion
SR: Sarcoplasmic reticulum
SERCA: Sarcoplasmic reticulum calcium ion -ATPase
PLN: Phospholamban
SUMO: Small ubiquitin related modifiers
PP1: Type I protein phosphatase
I1c: Constitutively active form of inhibitor 1
cAMP: Adenosine 3’,5’-monophosphate
AC: Adenylyl cyclase
PKP2: Plakophilin-2
ARVC: Arrhythmogenic right ventricular cardiomyopathy
miRNA: MicroRNA
modRNA: Modified mRNA
Ad: Adenovirus
AAV: Adeno-associated virus
LV: Lentivirus
Had5: Human adenovirus serotype 5
LAD: Left anterior descending artery
DRP: DNase-resistant particles
MACE: Major adverse cardiac events
LVEF: Left ventricle ejection fraction
RCT: Randomized controlled trial
RCSI: Retrograde coronary sinus infusion
ATTRv-PN: Transthyretin amyloidosis with polyneuropathy
ATTR-CM: Transthyretin amyloidosis-related cardiomyopathy
TTR: Transthyretin
